# The biosynthetic pathway of coumarin and its genetic regulation in response to biotic and abiotic stresses

**DOI:** 10.3389/fpls.2025.1599591

**Published:** 2025-06-19

**Authors:** Yixue Wang, Tiqing Guan, Xianling Yue, Jiashuo Yang, Xiaomin Zhao, Aixia Chang, Changqing Yang, Zhenjun Fan, Keqiang Liu, Yiting Li

**Affiliations:** ^1^ Tobacco Research Institute, Chinese Academy of Agricultural Sciences, Qingdao, China; ^2^ The Graduate School, Chinese Academy of Agricultural Sciences, Beijing, China; ^3^ Technology Center, Heilongjiang Tobacco Industry Limited Liability Company, Harbin, China; ^4^ Hunan Tobacco Corporation, Changsha, China

**Keywords:** coumarin, biosynthetic pathway, phenylpropanoids, environmental stress, genetic regulation

## Abstract

Coumarins, a class of metabolites derived from the phenylpropanoid pathway, play critical roles in plant development and interactions with environmental factors. In recent years, numerous studies have revealed that catalytic enzymes, physiological conditions, and environmental stimuli collectively regulate coumarin metabolism in plants. This regulation is not only essential for normal growth and development, but also enhances plant resistance to environmental stresses. In this review, we summarize recent advances in understanding the roles of coumarins in plant development, the key enzymes and genes involved in their biosynthesis, and the genetic regulatory mechanisms that mediate plant responses to both biotic and abiotic stresses, including drought, salinity, UV radiation, and attacks by pathogenic bacteria and insects. The strategic implementation of multi-gene regulatory approaches holds great promise for enhancing plant stress tolerance and has significant potential applications in agriculture.

## 1 Introduction

Coumarins constitute a special family of secondary metabolites in plants. To date, a total of 574 coumarins, reported from plant sources, have been enumerated. These coumarins are distributed over nearly 30 families and 150 species, such as *Umbelliferae*, *Apiaceae*, *Asteraceae*, *Fabaceae*, *Moraceae*, *Oleaceae*, *Rutaceae*, *Thymelaeaceae*, and so on (Leal et al., 2024; Nan et al., 2025). The discovery of natural products of coumarin dates back two centuries, with the nomenclature originating from the plant *Coumarouna odorata* (*Dipteryx odorata*) (Barot et al., 2015; Pereira et al., 2018; Annunziata et al., 2020). Subsequent investigations have isolated and characterized structurally diverse coumarins and their derivatives from various plant tissues, such as roots, leaves, fruits, and flowers, and discovered their broad-spectrum bioactivities and multifaceted application prospects in the fields including pharmaceutical and healthcare industries, fragrance production, and sustainable agricultural practices (Wu et al., 2009; Hussain et al., 2019; Nan et al., 2025).

So far, the main biosynthetic pathways of coumarins in plants have been well-characterized, encompassing an upstream phenylpropanoid pathway and a downstream coumarin-specific branch pathway. These processes involve a suite of catalytic enzymes, including phenylalanine ammonia-lyase (PAL), cinnamate 4-hydroxylase (C4H) and 4-coumarate-CoA ligase (4CL), which catalyzed the initial three steps of the pathway and provide the basis for coumarins branch, as well as coumarate 2’-hydroxylase (C2’H), p-coumaroyl shikimate 3’-hydroxylase (C3H), and feruloyl-CoA 6’-hydroxylase (F6’H), prenyltransferases (PTs) and cytochrome P450 monooxygenases, which were responsible for the formation of simple and complex coumarin derivatives. Up to now, *Apiaceae* is the only lineage in which the complete biosynthetic pathway has been decoded (Thomas, 2010; Huang et al., 2024; Liang et al., 2024; Goldenberg et al., 2025). Notably, the homeostasis of coumarins in plants is dynamically regulated by biotic and abiotic stresses, reflecting an adaptive mechanism evolved by plants to cope with intricate and fluctuating environments. The biosynthesis and accumulation of coumarins in plants are tightly regulated by a combination of abiotic factors (e.g., nutrient availability, water status, temperature, and light conditions) and biotic stressors (e.g., pathogenic microorganisms and herbivorous pests) (Duan et al., 2019; Robe et al., 2020; Huang et al., 2022; Bai et al., 2024).

An in-depth understanding of the biosynthesis of coumarins and the molecular regulatory mechanisms under diverse stress conditions will extend our knowledge of plant physiological and ecological processes. It will offer solid theoretical and practical guidance for crop breeding, agricultural production, and biomedical research. This review outlines the regulatory roles of enzymes in coumarin metabolism, their contributions to coordinating plant development and plant-environment interactions, and the molecular mechanisms driving coumarin accumulation in response to biotic and abiotic stresses. We also highlight the current research gaps in applying these findings to breeding and agriculture, proposing future research directions for improving plant stress resistance through genetic engineering methods.

## 2 The structure and functions of coumarins

### 2.1 Structural composition of coumarins

**Figure 1:**
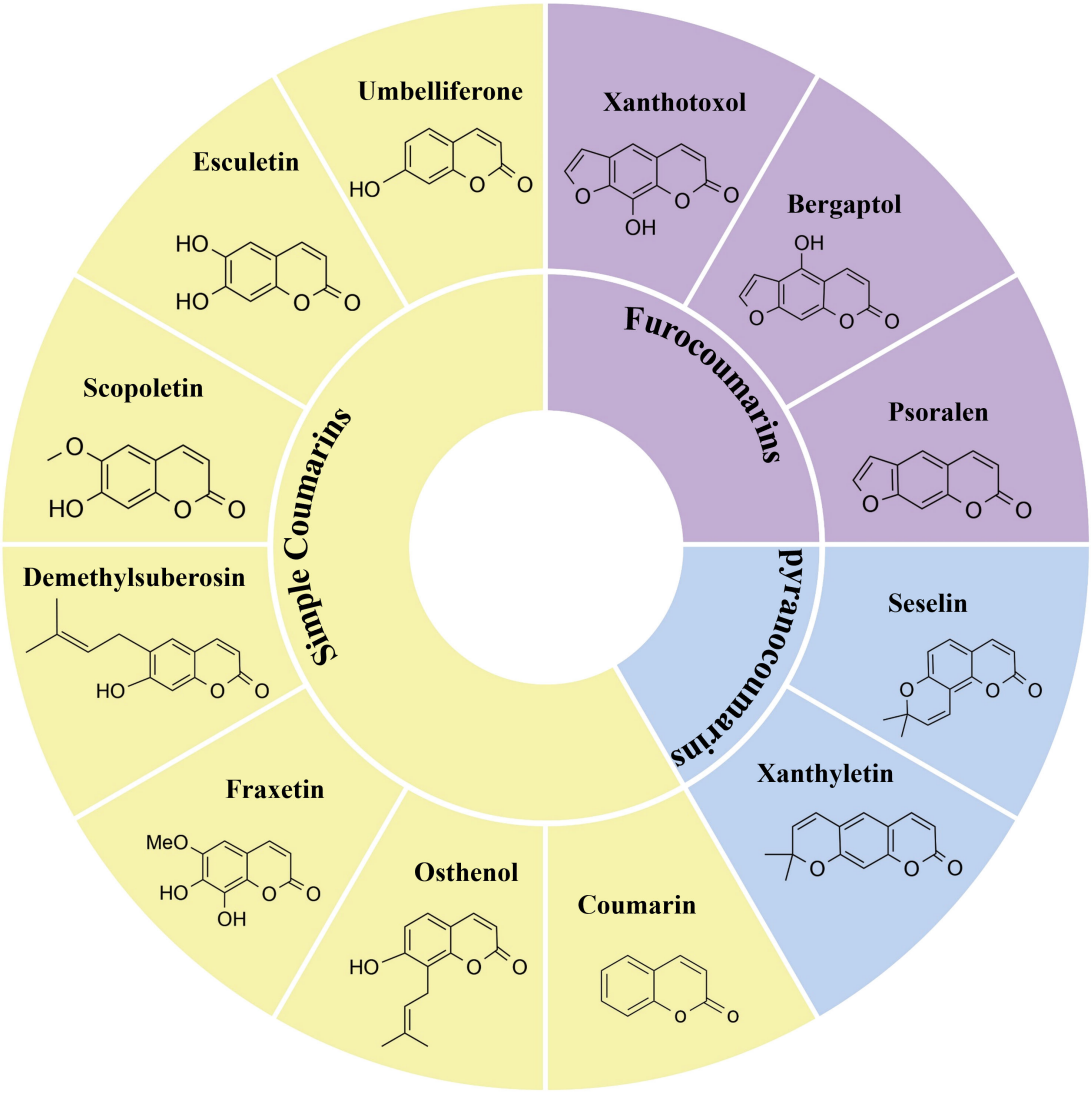
Examples of simple and composite coumarins. Coumarin, osthenol, fraxetin, demethylsuberosin, scopoletin, esculetin, umbelliferone are Simple coumarins. Psoralen, bergaptol and xanthotoxol are furocoumarins. Seselin and xanthyletin are pyranocoumarins.

Coumarin constitutes an important class of phenolic compounds and a mid-size family of secondary metabolites in plants. Common examples include scopoletin (7-hydroxy-6-methoxy-coumarin), umbelliferone (7-hydroxy-coumarin), and esculetin (6,7-dihydroxy-coumarin), etc (Leal et al., 2024). Structurally, coumarins consist of a benzene ring that is fused with an α-pyrone ring, forming a benzopyrone structure. Based on their structural characteristics, coumarins are classified into simple and composite coumarins (Sierra et al., 2021; Rohman et al., 2024). Simple coumarins are characterized by a 1,2-benzopyrone core, substituted on the benzene ring with hydroxyl, methoxy, methylenedioxy, or isopentenyl groups, and lacking fused heterocyclic systems (e.g., furan or pyran rings) formed via linkages between the C-7 hydroxyl group and C-6/C-8 positions. For instance, esculetin, which exists in *C. fraxini*, and the scoparone isolated from *A. capillaris* (Zhu and Jiang, 2018; Annunziata et al., 2020). The specific arrangements of tetrahydropyran (THP) and tetrahydrofuran (THF) rings with lactone structures give rise to at least four types of composite coumarins: linear furan coumarins, angular furan coumarins, linear pyranocoumarins, and angular pyranocoumarins. Through dehydration condensation between the C-7 hydroxyl group on the benzene ring and adjacent C-6 or C-8 positions, simple coumarins are converted into furanocoumarins, forming linear (e.g., psoralen) or angular (e.g., isopsoralen) subtypes fused with a furan ring. Pyranocoumarins are formed through cyclization between a hydroxyl or methoxy group at positions such as C-5 or C-6 and adjacent carbons (e.g., C-4 or C-7) on the benzene ring, generating a fused pyran ring, and are categorized into linear (e.g., xanthyletin) or angular (e.g., seselin) subtypes based on the spatial orientation of the pyran ring (Zhu and Jiang, 2018; Annunziata et al., 2020) (**Figure 1**).

### 2.2 Functions of coumarins

The structural diversity of coumarins endows them with a wide range of biological activities, thus making them important lead compounds in drug discovery and development (Nan et al., 2025). As a kind of natural product of plants, coumarin and its derivatives exhibit diverse therapeutic applications such as anti-human immunodeficiency virus (HIV) therapy, antitumor, antioxidant, antimicrobial, anti-inflammatory analgesic, anti-coagulant activities (Zhu and Jiang, 2018; Sierra et al., 2021; Zeng et al., 2024). In the 1990s, the tetracyclic pyranocoumarin compounds calanolide A and calanolide B, isolated from *Calophyllum lanigerum*, were first discovered to exhibit potent inhibitory activity against HIV replication, becoming the first anti-HIV plant extract to enter clinical trials (Kashman et al., 1992). Subsequently, a large number of coumarin structurally modified derivatives with increased anti-HIV activity gradually emerged, such as psoralen, bergapten, imperatorin, suksdorfin, byakangelicin, and 3-phenylcoumarins. The widely used warfarin (4-hydroxy-3-(3-oxo-1-phenylbutyl) coumarin, acenocoumarol, and phenprocoumon exert anticoagulant properties by inhibiting the vitamin K epoxide reductase complex (Zhu and Jiang, 2018; Hussain et al., 2019). New coumarin-thiazolidinone and/or thiazole conjugates can treat tuberculosis (TB) by inhibiting the InhA enzyme (Ebaid et al., 2025). Osthole can trigger cAMP/PKA-dependent relaxation of airways to induce bronchodilation without desensitizing receptors or increasing the risk of death (Wang et al., 2020).

Coumarin not only in terms of human health, but also shows great application prospects in green agricultural production, including regulating plant growth, serving as phytoalexin, insecticide, and bacteriostatic agent. For example, furanocoumarins (FCs), a subgroup of coumarins, are considered as natural insecticides and fungicides because they can prevent the invasion of plant-pathogenic microorganisms and the predation of herbivorous insects (Shao et al., 2022). *Bituminaria bituminosa* (*L.*) accumulates high concentrations of furanocoumarins, such as angelicin and psoralen, which help the plant resist infection and herbivore attack (Walker et al., 2012). Coumarins can delay seed germination by reducing endogenous GA4 levels, thereby decreasing the accumulation of Reactive Oxygen Species (ROS) (Chen et al., 2021). Most 7-vinylcoumarin derivatives exhibit good inhibitory activity against *Colletotrichum gloeosporioides* (Zhang et al., 2025). Some of coumarin derivatives containing the 1,3,4-oxadiazole/thiadiazole moiety exhibited good *in vivo* antiviral efficacy against tobacco mosaic virus (TMV) (Hu et al., 2025). *Phakopsora pachyrhizi (Pp)* can cause Asian soybean rust (SBR) disease. Scopoletin has action of antibacterial activity against *Pp*, which is related to the reduction of the accumulation of reactive oxygen species (ROS) in the fungal pre-infection structure (Beyer et al., 2019).

Due to its sweet aroma, coumarin has been extensively utilized in perfumery since its discovery in the 19th century. In violet-type scents, coumarin imparts the quintessential “hay-like” and “sweet” olfactory profile, thereby becoming a defining component in modern perfumery. As a fragrance enhancer, coumarin can be used in products such as soaps, detergents, and cosmetics to mask unpleasant odors and endow them with a soft and sustained fragrance (Camillo et al., 2023).

## 3 Coumarin synthesis pathway and its transcriptional regulation

### 3.1 General pathway

**Figure 2:**
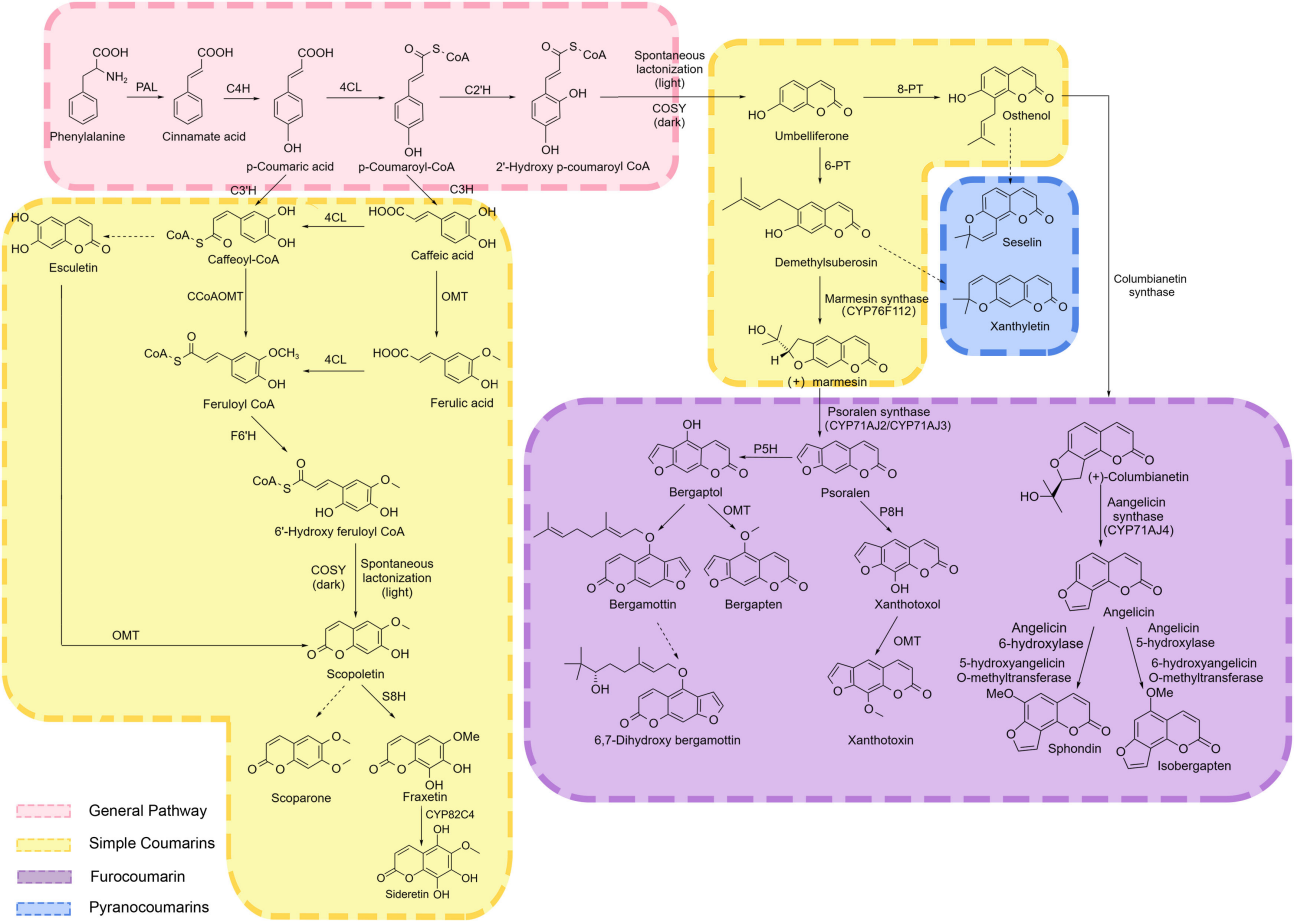
Coumarin synthesis pathway. PAL, phenylalanine ammonia-lyase; C4H, cinnamic acid 4-hydroxylase; 4CL, 4-coumarate-CoA ligase; C2’H, Cinnamate-2-hydroxylase; C3’H, Coumarate-3-hydroxylase; CCoAOMT, Caffeoyl-CoA-O-methyltransferase; F6’H, Feruloyl-CoA 6‘-hydroxylase; COSY, Coumarin synthase; S8H, Scopoletin 8-hydroxylase; PT, prenyltransferase; P5H, psoralen 5-hydroxylase; P8H, psoralen 8-hydroxylase; OMT, O-Methyltransferases. Black arrows indicate single direct reaction, dashed black arrows represent unauthenticated steps. General pathway metabolites are indicated by pink background. Simple Coumarins biosynthesis pathway metabolites are indicated by yellow background. Furococoumarin biosynthesis pathway metabolites are indicated by purple background. Pyranocoumarins biosynthesis pathway metabolites are indicated by blue background.

The phenylpropanoid pathway in plants is one of the most important and highly conserved biosynthetic pathways of secondary metabolites. It starts with phenylalanine, which is synthesized via the glycolysis and the shikimate pathway. In a three-step process catalyzed by phenylalanine ammonia-lyase (PAL), cinnamic acid 4-hydroxylase (C4H), and 4-coumarate-CoA ligase (4CL), cinnamic acid, p-coumaric acid, and p-coumarate-CoA are produced sequentially (Thomas, 2010) (**Figure 2**). These metabolites serve as common precursors for the downstream metabolic processes, generating a diverse array of secondary metabolites, including lignin, flavonoids, coumarins, phenolics, and organic acids, forming a complex and multi-branched phenylpropanoid biosynthetic pathway. Among these, the lignin and flavonoid synthesis pathways are the most prominent ones, whereas the coumarin biosynthetic pathway is another essential branch of the phenylpropanoid pathway (Dong and Lin, 2021). The transcriptional activation of the upstream phenylpropanoid pathway often triggers a metabolic reprogramming cascade in the downstream networks (Chen et al., 2023). For instance, overexpression of the *C4H* gene exhibited corresponding increases in C4H enzymatic activity, which enhanced the biosynthesis of pyranocoumarin decursinol angelate in *Angelica gigas* hairy roots (Park et al., 2012).

### 3.2 Coumarin branching pathway

#### 3.2.1 Simple coumarin synthesis

The ortho-hydroxylation of cinnamic acid derivatives, leading to the formation of the coumarin lactone ring, is recognized as a critical step in coumarin biosynthesis. Ruolan et al. isolated a putative ortho-hydroxylase, p-coumaroyl CoA 2’-hydroxylase (C2’H), from *P. praeruptorum* Dunn, which exhibited the highest transcriptional activity in roots. Prokaryotic expression assays confirmed that PpC2’H catalyzes the conversion of p-coumaroyl-CoA to a hydroxylated intermediate, followed by spontaneous lactonization under light conditions or COSY-catalyzed lactonization in darkness to generate umbelliferone (Ruolan et al., 2017). In *Arabidopsis thaliana*, the biosynthesis of umbelliferone proceeds through the following sequential steps. CCoAOMT1, exhibiting 3’-O-methyltransferase activity, catalyzes the conversion of caffeoyl-CoA to feruloyl-CoA. The T-DNA insertion mutants of CCoAOMT1 displayed a significant reduction in scopoletin and scopolin levels in the roots. Feruloyl-CoA 6’-hydroxylase 1 (F6’H1) subsequently hydroxylates feruloyl-CoA to generate 6’-hydroxyferuloyl-CoA, which is partially spontaneously converted to scopoletin. This spontaneous process is significantly enhanced by the enzymatic activity of coumarin synthase (COSY), thereby optimizing scopoletin biosynthesis efficiency (Kai et al., 2008). Functional analysis of T-DNA mutants identified CYP98A3 as the key enzyme catalyzing the 3’-hydroxylation of p-coumarate in *A. thaliana*. A 97% reduction in scopoletin and scopolin content was observed in the mutant roots, suggesting the critical role of CYP98A3 in scopoletin and its derivatives biosynthesis (Kai et al., 2006). COSY catalyzes the biosynthesis of umbelliferone, esculetin, and scopoletin from their respective ortho-hydroxycinnamoyl-CoA thioester precursors through two sequential reaction steps: trans-cis isomerization followed by lactonization (Vanholme et al., 2019). C2’H and F6’H are two types of 2-oxoglutarate-dependent (2-OGD) dioxygenase family. Goldenberg et al. demonstrated that single dual specificity C2’H/F6’H directs FCs production in citrus leaves and fruit. F6’H only catalyzes the synthesis of scopoletin products, while C2’H catalyzes the synthesis of umbelliferone products (Goldenberg et al., 2025). Scopoletin 8-hydroxylase (S8H), a 2-oxoglutarate-dependent dioxygenases (2-ODDs), catalyzes the hydroxylation of scopoletin at the C-8 position, mediating its conversion to fraxetin. Fraxetin is further oxidized by the CYP enzyme (CYP82C4) to produce sideretin (Rajniak et al., 2018; Siwinska et al., 2018).

Glycosylation, a key modification in coumarin biosynthesis, is mediated by UDP-glycosyltransferases (UGTs) in the cytoplasm, thereby enhancing the solubility, stability, and bioactivity of coumarins. Among the 189 *MaUGT* genes identified in the *Melilotus albus* genome, 16 exhibited differential expression between the low-coumarin-content genotype (Ma44) and high-coumarin-content genotype (Ma49), suggesting their potential involvement in coumarin biosynthesis. Further heterologous expression assays confirmed that *MaUGT186* is responsible for the glycosylation of scopoletin (Duan et al., 2021). The gene cluster composed of six β-glucosidase (BGLU) genes contributes to the evolution of the coumarin biosynthesis pathway in *Melilotus albus*. *MaBGLU1* was confirmed to be involved in scopoletin (coumarin derivative) synthesis (Wu et al., 2022). The tobacco glucosyltransferase TOGT has been identified as a highly active enzyme capable of catalyzing the glucosylation of p-hydroxycoumarin and hydroxycinnamic acids. TOGT-depleted tobacco plants exhibited diminished UGT activity toward scopoletin, and a significant reduction in the accumulation of glucoside form of scopoletin (scopolin). Taguchi et al. cloned the cDNA encoding GTase (NtGT2) in *Nicotiana tabacum* L. and found that the recombinant enzyme (rNTGT2) expressed in *Escherichia coli* exhibited glucosylation activity toward 3-hydroxycoumarin (Fraissinet-Tachet et al., 1998; Chong et al., 2002; Taguchi et al., 2003). The glycosyltransferase AdCGT isolated from *Angelica sinensis* uses 5,7-dihydroxycoumarin as a substrate to produce a C-glycosylated product at C-8 position (Wang et al., 2022).

#### 3.2.2 Synthesis of composite coumarins

Umbelliferone serves as a precursor for the biosynthesis of other coumarins and as an entry point for furanocoumarins biosynthesis. Downstream of umbelliferone, multiple prenyltransferases (PTs) and cytochrome P450 family members are predicted to participate in the biosynthesis of furanocoumarins (Goldenberg et al., 2025). PTs are the main determinants of the structural diversity of composite coumarins (CCs). Umbelliferone 6/8-isopentenyltransferase promotes the substitution of umbelliferone by an isopentenyl group at the 6 or 8 position to form demethylsuberosin (DMS) or osthenol, respectively. This step is the starting point for the synthesis of composite coumarins and determines whether angular or linear coumarins are formed (Huang et al., 2024; Li et al., 2024). In *Pastinaca sativa*, PsPT1 and PsPT2 use umbelliferone and dimethylallylpyrophosphate (DMAPP) as substrates to target plastids and synthesize osthenol and demethylsuberosin (DMS) (Munakata et al., 2016). In *Petroselinum crispum*, PcPT exhibits strict substrate specificity toward umbelliferone and dimethylallyl diphosphate, shows a preference for acting on the C-6 position of the prenylated product (demethylsuberosin) to generate linear furanocoumarins. Only a small amount of the C8-prenylated derivative (osthenol) is produced (Karamat et al., 2014) (**Figure 2**).

Cytochrome P450s play key roles in the biosynthesis and hydroxylation of furanocoumarins, allowing the utilization of psoralen as a new substrate for the synthesis of bergamot alcohol. CYP76F112 acts as marmesin synthase (MS) that converts DMS to (+)-marmesin. Psoralen synthase (PS) converts (+)-marmesin to psoralen (Romain et al., 2007; Villard et al., 2021; Rodrigues et al., 2022). Psoralen is hydroxylated at the 5- and 8-positions by psoralen 5-hydroxylase (P5H) and psoralen 8-hydroxylase (P8H) to produce bergaptol and xanthotoxol. These products are then further converted to bergapten lactone and xanthotoxin by the corresponding O-methyltransferases (**Figure 2**) (Zhao et al., 2016, 2019; Zhang et al., 2022; Bouillé et al., 2025). CYP71AJ2 and CYP71AJ3 exhibited psoralen synthase activity and were responsible for converting marmesin into psoralen. They promote the synthesis of linear furanocoumarins by catalyzing this reaction. CYP71AJ4 has angelicin synthase activity and can catalyze the conversion of columbianetin into angelicin. Angelicin is converted into sphondin under the action of angelicin 6-hydroxylase and 5-hydroxyangelicin O-methyltransferase. It is transformed into isobergapten under the effect of angelicin 5-hydroxylase and 6-hydroxyangelicin O-methyltransferase. CYP71AJ4 enables the smooth synthesis of angular furanocoumarins (Larbat et al., 2009; Roselli et al., 2017).

Seselin and xanthyletin are two pyranocoumarins whose biosynthetic precursors are osthenol and demethylsuberosin, respectively, although their exact biosynthetic pathways have not yet been experimentally validated (Goldenberg et al., 2025).

### 3.3 Transcriptional regulation in the coumarin synthesis pathway

The co-expression network and cis-element analysis of the promoters revealed that differentially expressed MYB, bHLH, AP2, and WRKY transcription factors may play crucial roles in regulating the expression of coumarin biosynthetic genes (Chen et al., 2023). The MYB superfamily plays a critical role in plant growth, development, defense against environmental stress, and the biosynthesis of secondary metabolites. In *Peucedanum praeruptorum* Dunn, the expression patterns of R2R3-MYB transcriptional factors PpMYB3 and PpMYB103 in roots exhibited a strong spatiotemporal correlation with coumarins (including praeruptorin A, praeruptorin B, praeruptorin E, scopoletin, and isoscopoletin) accumulation profiles, suggesting their potential roles as positive transcriptional regulators of coumarins biosynthesis (Liao et al., 2024). Furanocoumarins serve as the primary bioactive constituents in *Angelica dahurica* var. *formosana* (ADF), which belongs to the *umbelliferae* family. These compounds are predominantly localized within the oil tubes of the root phloem of ADF. Functional studies revealed that overexpression of the *AdNAC20* transcription factor in ADF resulted in a 9.28% reduction in total coumarin content, accompanied by a significant 12.28% increase in lignin accumulation. Conversely, *AdNAC20*-knockout mutants exhibited a 16.3% elevation in total coumarins and a substantial 33.48% decrease in lignin levels. Confirming the dual-pathway regulatory effect of AdNAC20, that is, positively enhancing lignin formation but negatively controlling coumarin formation (Qu et al., 2024). The MYB72 transcription factor has been found to be involved in regulating the biosynthesis of scopoletin (including β-glucosidase BGLU42), whereas BGLU42 mediates scopoletin secretion into the rhizosphere by modifying its precursor (Lundberg and Teixeira, 2018). In *Gossypium hirsutum*(cotton), *GhF6’H1*, which is regulated by GhWRKY33, regulates the accumulation of scopoletin (Gao et al., 2024). Moreover, in *Nicotiana tabacum*, the ERF transcription factor *WAX INDUCER1* (*NtWIN1*) inhibits the accumulation of scopoletin through the activity of NtF6’H1 (He et al., 2024).

## 4 Molecular regulations of coumarin metabolic pathways in response to abiotic stress

### 4.1 Molecular regulation in response to nutrient deficiency

The interaction of soil microorganisms with coumarins participates in plant nutrient metabolism, which represents a crucial step in enhancing plant resistance to nutrient deficiencies. Low nitrogen promotes the secretion of carbon-containing compounds into the rhizosphere, substantially altering the composition of the rhizosphere bacterial community and stimulating the growth of *Angelica dahurica* and the synthesis of furanocoumarins (Jiang et al., 2024). Under nutrient-deprived conditions, inoculation with the plant growth-promoting rhizosphere bacteria (PGPR) strain *Bacillus velezensis* SAAS-63 reshaped rhizosphere metabolism by reprogramming phenylpropanoid pathway flux. The inoculant suppressed flavone and isoflavone biosynthesis while redirecting phenylalanine toward the synthesis of lignin precursors and coumarin-derived metabolites, which collectively alleviated nutrient deficiencies and enhanced plant resistance in lettuce (Bai et al., 2024).

Under iron-deficiency conditions, the expressions of *S8H* and *CYP82C4* were upregulated, accompanied by a large accumulation of coumarins in root secretions, including scopoletin, rehmanin, and astragalus in *A. thaliana* (Murgia et al., 2011; Rajniak et al., 2018). During *A. thaliana* growth and development, the secretion of esculetin, scopoletin, isoephedrine, and methoxycoumarins can be selectively enhanced by buffered nutrient solutions at pH 5.5 or 7.5. At the same time, the expression of *F6’H1* increased 4-fold and 8-fold at pH 5.5 and pH 7.5, respectively. The expression of *COMT* and *CCoAMT* genes in roots increased only at pH 7.5 under iron-deficient conditions (Sisó-Terraza et al., 2016). The peptides IRONMAN (IMA) work in concert with the environmental pH. By regulating the expression of key genes such as *MYB72, S8H* and *CYP82C4*, it alters the biosynthesis and secretion of coumarins (such as fraxetin and sideretin), optimizes the iron absorption process, and helps plants adapt to the iron nutritional status in different acidic and alkaline environments (Gautam et al., 2021). Recently, the ABC transporter G family member PDR9 has been reported to affect the coumarin accumulation and homeostasis under combined Fe and P deficiency. MYB63 transcription factor controls dedicated coumarin production by regulating both *COSY* and *F6’H1* expression while orchestrating secretion through *PDR9* genes under Fe and P combined deficiency (Fourcroy et al., 2014; DeLoose et al., 2024). *Nicotiana tabacum* secretes coumarins in response to iron deficiency. NtPDR3/NtABCG3, which is a plasma-membrane ABC transporter belonging to the pleiotropic drug resistance (PDR) family in *Nicotiana tabacum*, plays a crucial role in mediating secretion of O-methylated coumarins to the rhizosphere (Lefèvre et al., 2018) (**Table 1**).

**Table 1:** Effect of abiotic stress on coumarins variation in plants.

Abiotic Stress	Plants	Coumarins Composition or Levels Under Abiotic Stress	Reference
Nutrient Deficiency (Low nitrogen)	*Angelica dahurica* var. *Formosana*	The synthesis of furanocoumarins is increased.	(Jiang et al., 2024)
	Lettuce	The synthesis of coumarin-related is increased.	(Bai et al., 2024)
Nutrient Deficiency (Iron-deficient)	*Arabidopsis thaliana*	The levels of simple coumarins (scopoletin, esculetin) are elevated.	(Murgia et al., 2011; Rajniak et al., 2018)
		The expression of *F6‘H1*, *COMT* and *CCoAMT* are increased.	(Sisó-Terraza et al., 2016)
		The fraxetin and sideretin biosynthesis are altered by IRONMAN (IMA) peptides regulating MYB72, S8H and CYP82C4.	(Gautam et al., 2021)
		The coumarin accumulation is regulated by PDR9 and MYB63.	(Fourcroy et al., 2014; DeLoose et al., 2024)
	*Nicotiana tabacum*	The scretion of O-methylated coumarins is increased via NtPDR3/NtABCG3 transporter.	(Lefèvre et al., 2018)
Drought Stress	*Ficus deltoidea*	The coumarin content is increased.	(Manurung et al., 2019; Zhong et al., 2021)
	*Melilotus albus*	The content of coumarin in transgenic hairy roots is improved because expression of *MaCYP82L1* is increased.	(Nie et al., 2024)
		MaUGT68 and MaUGT186 enhance drought tolerance via coumarin glycosylation	(Duan et al., 2021)
	*Pinus sylvestris*	The transcriptional levels of *CYP71AJ49* and CYP71AJ51 (psoralen synthases and angelicin synthases) are downregulated.	(Jian et al., 2020)
Salt Stress	*Melilotus albus*	The content of coumarin in transgenic hairy roots is improved because expression of *MaCYP82L1* is increased.	(Nie et al., 2024)
	Soybean	The expression of GmF6‘H1 is increased.	(Duan et al., 2019)
Light and Darkness (90% shade condition)	*Angelica sinensis*	The expression levels of *4CL* and *COMT* are significantly down-regulated.	(Huang et al., 2022)
Light and Darkness	*Petroselinum crispum (parsley)*	The furanocoumarin production and PcPT expression are increased.	(Karamat et al., 2014)
Light and Darkness (under ultraviolet B radiation and darkness treatment)	*Clematis terniflora*	The coumarin biosynthesis is promoted by *CtCOSY.*	(Tao et al., 2023)
Cold Stress	*Arabidopsis thaliana*	The accumulation of scopoletin is decreased in the leaves because of a lesion in the THO1 homolog.	(Döll et al., 2018)

### 4.2 Molecular regulation in response to drought stress

Drought stress has been demonstrated to induce marked accumulation of coumarins in plants. *Ficus deltoidea* leaf extracts exhibit a 2.3-fold increase in total coumarin content under water deficit conditions (Manurung et al., 2019; Zhong et al., 2021). In *Melilotus albus*, *MaCYP82L1* is an important regulator of drought and salt resistance. Yeast cells transformed with *MaCYP82L1* exhibited significant resistance to drought and salt stress. Moreover, overexpression of *MaCYP82L1* led to an increase in the content of coumarin in transgenic hairy roots, laying a foundation for exploring the connection between coumarin metabolism and abiotic stresses (Nie et al., 2024). Two CYP71AJ enzymes (psoralen synthase and angelicin synthase) are involved in the biosynthesis of furanocoumarins in *Pinus sylvestris*. Under drought conditions, the transcriptional levels of both *CYP71AJ49* and *CYP71AJ51* are significantly downregulated (Jian et al., 2020). UDP-glycosyltransferases (UGTs) are responsible for the glycosylation modification of coumarin (Nie et al., 2024). UGT expression is significantly upregulated under drought stress, increasing coumarin biosynthesis and promoting adaptation to abiotic stress. *MaUGT68* and *MaUGT186* in the genome of *Melilotus albus* have effects on the growth and stress (drought and salt) resistance of yeast cells. The transformed yeast cells carrying *pYES2-MaUGT68* and *pYES2-MaUGT186* constructs showed remarkable resistance to 30% PEG-6000 treatment, especially at a 105-fold dilution, indicating that *MaUGT68* and *MaUGT186* enhance drought tolerance in yeast (Duan et al., 2021) (**Table 1**).

### 4.3 Molecular regulation in response to salt stress

Salt stress plays an important role in the regulation of coumarin biosynthesis. In *Melilotus albus*, expression of *MaCYP82L1* was also increased in response to salt treatment, and its heterologous expression in yeast led to significantly increased resistance to drought and salt stresses (Nie et al., 2024). Salt stress significantly induced the expression of *GmF6’H1* in soybean. When *GmF6’H1* was constitutively expressed in *A. thaliana* under the control of the 35S promoter, a significantly increased salt tolerance was observed (Duan et al., 2019) (**Table 1**).

### 4.4 Molecular regulation in response to light and darkness

The influence of light on the biosynthetic accumulation of coumarins might be mediated through the modulation of primary metabolic processes associated with photosynthesis, particularly carbon and nitrogen metabolism, which serve as fundamental sources of carbon skeletons and nitrogen precursors for coumarins synthesis (Ruan et al., 2010; Wu et al., 2023). By analyzing the differentially expressed genes (DEGs) related to coumarin biosynthesis in *Angelica sinensis* under 50%, 70%, and 90% shade conditions, researchers found that the expression levels of *4CL* and *COMT* were significantly down-regulated at 90% shade conditions (Huang et al., 2022). Ultraviolet (UV) irradiation will increase the expression of *PcPT* in various tissues of parsley (*Petroselinum crispum*), and at the same time, the production of furanocoumarins also increases (Karamat et al., 2014). The content of coumarins in *Clematis terniflora* leaves increased under ultraviolet B radiation and darkness treatment (UV-D). Under UV-D stress, *CtCOSY* influenced the biosynthesis of phenylpropanoid compounds in *C. terniflora*, promoting coumarin biosynthesis and affecting purine metabolism (Tao et al., 2023) (**Table 1**).

### 4.5 Molecular regulation in response to cold stress

Scopoletin accumulation was also detected in cells subjected to cold stress. The THO/TREX complex is a critical multi-protein complex involved in coupling transcription, mRNA processing, and nuclear export. In *Arabidopsis thaliana*, the *Tho1* mutant resulted from a lesion in the THO1 homolog encoding a THO/TREX complex subunit. This mutation led to a cold-induced decrease in the accumulation of scopoletin in the leaves. Mutations in genes encoding putative *Arabidopsis* THO/TREX complex subunits, aside from THO1, partially reduced the scopoletin content in roots under osmotic/high-carbon stress and in leaves under cold stress. Mutations in AGO1, RDR6, and SGS3, which are involved in the RNA silencing pathway, also affected the cold-induced accumulation of scopoletin in leaves (Döll et al., 2018) (**Table 1**).

## 5 Molecular regulations of coumarin metabolic pathways in response to biotic stresses

Coumarins and their derivatives play a crucial role in plant responses to biotic stress. They are involved in plant defense reactions against various pathogens, and their synthesis and accumulation are finely regulated by multiple factors. Numerous studies have shown that when plants face different biotic stresses, coumarin-related metabolic pathways undergo significant changes, thereby affecting plant disease resistance and growth and development (Dixon, 2001; Wu et al., 2023).

Scopoletin specifically inhibits soil-borne fungal pathogens such as *Fusarium oxysporum* and *Verticillium dahliae*. The plant and beneficial rhizobacteria work together to trigger MYB72/BGLU4-dependent scopoletin production and excretion, which in turn selectively inhibits these soilborne fungal pathogens (Stringlis et al., 2018). As a transcription factor, MYB72 may be a key regulator of the genes involved in scopoletin biosynthesis (including *BGLU42*). *BGLU42* modifies the precursors of scopoletin and mediates its secretion into the rhizosphere (Lundberg and Teixeira, 2018). In *Gossypium hirsutum*(cotton), *GhF6’H1*, regulated by GhWRKY33-like factors, plays a crucial role in enhancing cotton resistance to *Verticillium dahliae* by regulating scopoletin accumulation (Gao et al., 2024). AtBGLU40 belongs to a family of β-glucosidases with coumarin-hydrolyzing activity, and its enzyme activity is enhanced by *Fusarium oxysporum* infection (Rivera et al., 2024). The combined action of arbuscular mycorrhizal fungi (AMF) and elevated CO_2_ (eCO_2_) led to the accumulation of sucrose in the stems and roots of *Ammi majus*. This up-regulated the activity of *PAL*, induced the accumulation of coumarin, and enhanced the medicinal and pharmacological value of *Ammi majus* (Mohammed et al., 2022). After infection with *Alternaria alternata*, the jasmonate (JA) signaling pathway was activated in the leaves of *N. attenuata*. Subsequently, part of the regulation of scopoletin biosynthesis occurred through MYC2 to defend against *A. alternata*. The higher levels of scopoletin accumulated in young leaves contributed to their stronger resistance (Sun et al., 2014). LRR-RK4 was identified as the first leucine-rich repeat receptor-like kinase involved in the resistance to *Alternaria* in tobacco species, and it regulates *NaERF109* and *NaDEF19*. In virus-induced gene silencing (VIGS) plants with silenced *NaERF109*, the expression of *A. alternata*-induced *NaF6’H1* and *NaDEF19* was significantly reduced, which enhanced the susceptibility of NaLRR-RK4-RNAi lines to *A. alternata* (Zhao et al., 2022). After *Hyaloperonospora arabidopsidis* (Hpa) infection, *Arabidopsis thaliana* activates coumarin biosynthesis-related genes such as *MYB72* and *F6’H1*, inducing the generation of soil-borne legacy (SBL). Subsequently, this signal is transmitted through the salicylic acid (SA) pathway, enabling the plant to develop resistance against subsequent foliar downy mildew infections (Vismans et al., 2022). After being infected by *Erysiphe heraclei*, the overexpression of the *HmF6’H1* gene leads to an increase in the levels of simple coumarins. This overexpression inhibits the biosynthesis of furanocoumarins and pyranocoumarins by suppressing the expression of *PT* genes, thereby enhancing the resistance of *Heracleum moellendorffii* Hance to powdery mildew (Liu et al., 2024). The attack of thrips and *Echinothrips americanus* not only led to an increase in the content of GMCAs and herniarin, but also increased the amount of coumarin and umbelliferone (Repčák and Suvák, 2013). During pathogen invasion, the glycosyltransferase UGT73C7 catalyzes the formation of glucosinolates from coumaric acid and ferulic acid. This redirects the phenylpropane metabolism, leading to the accumulation of hydroxycinnamic acid and coumarin analogs, which stimulate the expression of the disease-resistance gene *snc1* and regulate plant immunity (Huang et al., 2021) (**Table 2**).

**Table 2:** Effect of biotic stress on coumarins variation in plants.

Biotic Stress	Crops	Coumarins Composition or Levels Under Biotic Stress	Reference
*Fusarium oxysporum*	*Arabidopsis thaliana*	The production and excretion of scopoletin are improved.	(Lundberg and Teixeira, 2018; Stringlis et al., 2018)
	Cotton(Pima-S6)	The enzyme activity of AtBGLU40 is enhanced.	(Rivera et al., 2024)
*Verticillium dahliae*	*Arabidopsis thaliana*	The production and excretion of scopoletin are improved.	(Lundberg and Teixeira, 2018; Stringlis et al., 2018)
	*Gossypium hirsutum*	The accumulation of scopoletin is increased.	(Gao et al., 2024)
*Arbuscular mycorrhizal fungi* (AMF)	*Ammi majus*	The activity of PAL is up-regulated, and the accumulation of coumarinis induced.	(Mohammed et al., 2022)
*Alternaria alternata*	*N. tabacum*	The accumulation of scopoletin is increased.	(Sun et al., 2014)
*Alternaria Alternating*	Tobacco	The expression of *NaF6’H1* and *NaDEF19* are decreased via the regulations of LRR-RK4.	(Zhao et al., 2022)
*Echinothrips americanus*	*Matricaria chamomilla*	The amount of coumarin and umbelliferone are increased.	(Repčák and Suvák, 2013)
*Hyaloperonospora Arabidopsidis* (HPA)	*Arabidopsis thaliana*	The genes such as *MYB72* and *F6’H1* are activated.	(Vismans et al., 2022)
*Erysiphe heraclei*	*Heracleum moellendorffii Hance*	The overexpression of the *HmF6’H1* gene leads to an increase in the levels of simple coumarins. And overexpression inhibits the biosynthesis of furanocoumarins and pyranocoumarins by suppressing the expression of *PT* genes.	(Liu et al., 2024)
Pthogen	*Arabidopsis*	The accumulations of hydroxycinnamic acid and coumarin analogs are increased.	(Huang et al., 2021))

## 6 Conclusions

Several genes, including *PAL*, *COMT*, *CCoAOMT*, *4CL*, *C3H*, *CSE*, and *C4H*, are involved in coumarin biosynthesis, and they exhibit tissue-specific expression patterns. Differentially expressed MYB, bHLH, AP2, and WRKY transcription factors have been found to play critical roles in regulating the expression of coumarin biosynthetic genes. However, compared to MYB transcription factors, there are relatively few studies on other transcription factors in the coumarin synthesis pathway. Moreover, compared to coumarins, the regulation of these transcription factors in the synthesis pathways of other phenylpropanoids (such as flavonoids and lignans) has been more extensively studied. Since these pathways belong to the same phenylpropanoid metabolism network as coumarin synthesis, they can provide valuable clues for studying the transcriptional regulation of the coumarin synthesis pathway.

Recent studies have shown that nutrient deficiency, drought, salinity, and light-dark changes can influence the expression of coumarin biosynthetic genes and induce coumarin accumulation. Plants rely on the antioxidant capacity of coumarin to help them cope with stress and adapt to the environment. Certain pathogenic microorganisms and insect infestations also induce coumarin accumulation, which is utilized by plants to defend against pests and diseases. Although numerous genes and genetic regulatory mechanisms related to coumarin synthesis and metabolism have been reported, most of these studies are applied in biomedical research. There are very few investigations into the genes that are practically relevant in breeding and agricultural production.

Coumarins often interact with other phenylpropanoids in response to biotic and abiotic stresses in plants, and the molecular regulation of these substances is connected. Therefore, the relationship between coumarin and other phenylpropanoid interactions and plant responses to biotic and abiotic stresses warrants further investigation. As the related enzyme genes in the coumarin anabolic pathway also affect the biosynthesis of other phenylpropanoids, genetic engineering may be used to conduct multi-gene combination regulation in the future. This approach aims to enhance plant stress tolerance and achieve practical applications in production.
